# The roles of calcium signaling and ERK1/2 phosphorylation in a *Pax6*^+/- ^mouse model of epithelial wound-healing delay

**DOI:** 10.1186/1741-7007-4-27

**Published:** 2006-08-16

**Authors:** Lucy J Leiper, Petr Walczysko, Romana Kucerova, Jingxing Ou, Lynne J Shanley, Diane Lawson, John V Forrester, Colin D McCaig, Min Zhao, J Martin Collinson

**Affiliations:** 1 School of Medical Sciences, Institute of Medical Sciences, University of Aberdeen, Aberdeen AB25 2ZD, UK

## Abstract

**Background:**

Congenital aniridia caused by heterozygousity at the *PAX6 *locus is associated with ocular surface disease including keratopathy. It is not clear whether the keratopathy is a direct result of reduced *PAX6 *gene dosage in the cornea itself, or due to recurrent corneal trauma secondary to defects such as dry eye caused by loss of *PAX6 *in other tissues. We investigated the hypothesis that reducing *Pax6 *gene dosage leads to corneal wound-healing defects. and assayed the immediate molecular responses to wounding in wild-type and mutant corneal epithelial cells.

**Results:**

*Pax6*^+/- ^mouse corneal epithelia exhibited a 2-hour delay in their response to wounding, but subsequently the cells migrated normally to repair the wound. Both *Pax6*^+/+ ^and *Pax6*^+/- ^epithelia activated immediate wound-induced waves of intracellular calcium signaling. However, the intensity and speed of propagation of the calcium wave, mediated by release from intracellular stores, was reduced in *Pax6*^+/- ^cells. Initiation and propagation of the calcium wave could be largely decoupled, and both phases of the calcium wave responses were required for wound healing. Wounded cells phosphorylated the extracellular signal-related kinases 1/2 (phospho-ERK1/2). ERK1/2 activation was shown to be required for rapid initiation of wound healing, but had only a minor effect on the rate of cell migration in a healing epithelial sheet. Addition of exogenous epidermal growth factor (EGF) to wounded *Pax6*^+/- ^cells restored the calcium wave, increased ERK1/2 activation and restored the immediate healing response to wild-type levels.

**Conclusion:**

The study links Pax6 deficiency to a previously overlooked wound-healing delay. It demonstrates that defective calcium signaling in *Pax6*^+/- ^cells underlies this delay, and shows that it can be pharmacologically corrected. ERK1/2 phosphorylation is required for the rapid initiation of wound healing. A model is presented whereby minor abrasions, which are quickly healed in normal corneas, transiently persist in aniridic patients, compromising the corneal stroma.

## Background

The transcription factor PAX6 is required for development and maintenance of several human ocular tissues, including the corneal epithelium. The gene *PAX6 *was identified by positional cloning as the defective gene in human aniridia and its associated ocular complications [1-3].

Heterozygous deficiency in *PAX6 *leads to ocular surface disease characterized by corneal opacification (aniridia-related keratopathy; ARK), and the *Pax6*^+/- ^mouse model displays all the morphologic defects of human ARK [4-7]. ARK in *Pax6*^+/- ^mice and humans is characterized by the presence of thin, fragile vacuolated corneal epithelia [4,6]. There is ingrowth of blood vessels into the cornea, sporadic appearance of goblet cells [8,9], and infiltration by inflammatory cells [10]. The cell biology that links *Pax6 *dosage to changes in cellular behavior remains poorly understood. Maintenance of the structural integrity of the corneal epithelium is essential for corneal transparency. Impaired re-epithelialization after wounding increases the risk of infection, exacerbates inflammation, and undermines normal stromal remodeling [11]. The cumulative effect of very small corneal scars following chronic failure of wound repair may result in loss of transparency. It has recently been suggested that ARK may be due to corneal epithelial fragility combined with an abnormal wound-healing response [12], resulting in degeneration of the cornea.

Epithelial sheets react to mechanical injuries via a complex process that relies on coordinated proliferation and migration into the denuded area, matrix deposition and tissue remodeling [13-16]. Wound-induced migration and proliferation are regulated by cytokines, extracellular matrix molecules, and growth factors including insulin-like growth factor, keratinocyte growth factor, hepatocyte growth factor and epidermal growth factor (EGF) [17-22].

One of the first reactions to injury of the cell monolayer is an intracellular calcium rise spreading as a wave from the injury site to the neighboring cells. This has been reported in a range of cell systems, mainly in cell lines [23-29]. The wound-elicited calcium wave stimulates cell proliferation after injury, and is required for further healing processes [24]. The intensity of the calcium response quantitatively controls the subsequent rate of healing in some epithelial cells, and remodeled calcium signaling may underlie disease states such as diabetes, hypertension, heart disease, manic depression and Alzheimer's disease [30-32].

Alongside the calcium wave, the other early response of epithelial cells to wounding is at least one slower wave of mitogen-activated protein kinase (MAPK) ERK1/2 phosphorylation [33]. The migration of at least some epithelial cell types appears to be ERK1/2-driven [34]. Addition of EGF activates ERK1/2 in a 'wild-type' rabbit corneal epithelial cell line [35], and exerts positive effects on epithelial wound healing by promoting migration and mitosis [17,29,36-38]. In corneal epithelial cells, EGF exhibits a positive effect on proliferation via orchestrated calcium influx from intracellular calcium stores and extracellular space [29,39].

Work by our laboratory and several others have investigated the roles of Pax6 in control of cell adhesion in development and disease, but the downstream cell-signaling pathways have received less attention. We hypothesised that cell signaling may be faulty. As a model for the reactive cellular pathways that may be controlled by Pax6, we have developed an *in vitro *wounding assay for murine primary corneal epithelial cells and have investigated the roles of *Pax6 *in corneal healing. We find that unlike wild-type, *Pax6*^+/- ^cells fail to commence healing within the first 2 hours after wounding, and that wound-induced waves of calcium and phospho-ERK1/2 signaling are required for rapid response to wounding. Propagation of the wound-induced calcium wave is defective in *Pax6*^+/- ^cells, but addition of EGF restores calcium signaling and promotes ERK1/2 phosphorylation, rescuing their rapid response to wounding.

## Results

### Wound healing is delayed in *Pax6*^+/- ^corneal epithelial cultures

**Figure 1:**
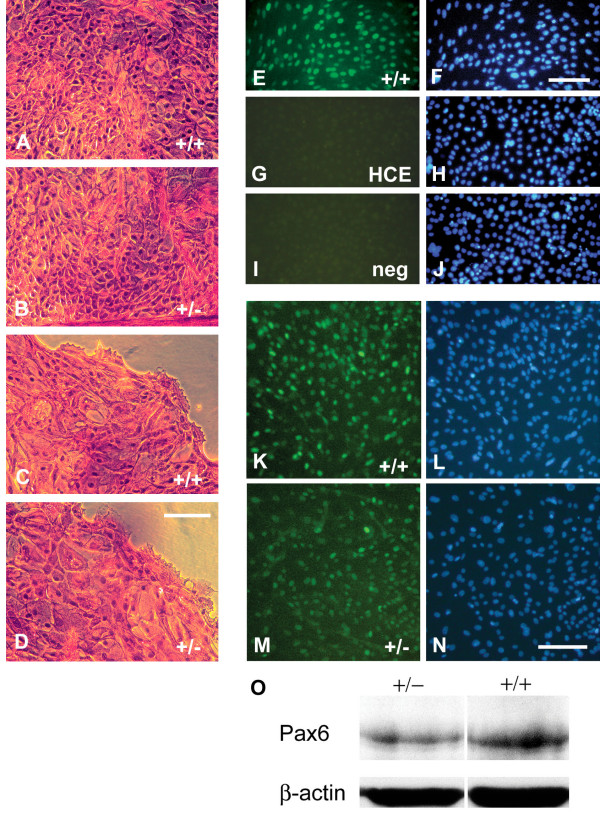
**Morphology and *Pax6 *expression of cultured corneal epithelial cells**. **(A) **Hematoxylin and eosin staining of confluent *Pax6*^+/+ ^and **(B) ***Pax6*^+/- ^corneal epithelial cell cultures. Both in confluence and at the outgrowing edge of new cultures **(C, D)**, the *Pax6*^+/- ^cells were morphologically indistinguishable from wild-type. **(E) **Immunocytochemistry on primary *Pax6*^+/+ ^mouse corneal epithelial cells compared with (**G**) the human corneal epithelial cell line, HCE. **(I) **Negative control. (**F**, **H**, **J**) DAPI-labeled cells. **(K, N) **Directly comparable Pax6 immunolabelling of *Pax6*^+/+ ^and *Pax6*^+/- ^corneal epithelial-cell cultures. (**L**, **N**) DAPI labeling. **(O) **Western blot of protein lysate from *Pax6*^+/+ ^and *Pax6*^+/- ^corneal epithelial cell cultures probed for Pax6 and β-actin. Scale bars = 100 μm.

The wound-healing response was investigated in primary murine corneal epithelial cells, cultured as described previously [40] on plastic or glass on which epithelial cells could attach and proliferate. All experiments were performed on mouse cells. As shown in Figure 1(A–D), *Pax6*^+/- ^and *Pax6*^+/+ ^cells looked morphologically identical, although *Pax6*^+/- ^cultures grew more slowly than wild-type in the first week after explant, and had multiple defects in cell-surface glycoproteins, which will be described elsewhere (RK, JO, LJL, JMC, manuscript submitted). Epithelial monolayers were strongly positive for nuclear Pax6 staining (Figure 1E), which is required for corneal epithelial development and is a definitive marker for these cells. In contrast, the widely used human corneal epithelial cell line (HCE) [29,41,42] was found not to express *PAX6 *(Figure 1G). *Pax6*^+/- ^cultures had lower levels of Pax6 protein than wild-type, assayed both by immunocytochemistry (Figure 1K–N) and Western blotting (Figure 1O). Densitometric analysis of the Western blots (subtracting background and dividing the intensity of Pax6 staining by that of β-actin) showed that *Pax6*^+/- ^cultures had 63% of the amount of Pax6 produced by wild-type cultures.

**Figure 2:**
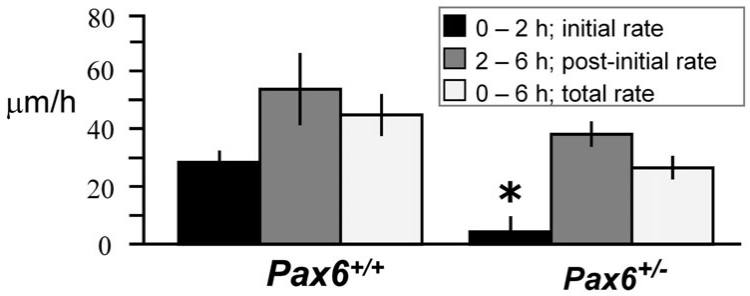
**Mean wound-healing rates for *Pax6*^+/+ ^and *Pax6*^+/- ^corneal epithelial cultures**. Wound healing is divided into an initial phase (0–2 hours, black bars), a post-initial phase (2–6 hours, dark grey) and a mean total over 0–6 hours (pale grey). The initial rate of wound healing in *Pax6*^+/- ^cells is significantly lower than that in wild-type (mean ± SEM, * *p *< 0.01).

Corneal epithelial cultures from *Pax6*^+/+ ^and *Pax6*^+/- ^littermates were injured as described in Methods and the rate of migration into the denuded area measured. All wounds healed, and the rates of cell migration were similar to those observed *in vivo *(20–50 μm/h) in wild-type rat corneas by Song et al [43]. However, *Pax6*^+/- ^cultures exhibited a wound-healing delay. Wound healing in the first 2 h after injury was negligible in *Pax6*^+/- ^cultures (mean ± SEM 4.4 ± 5.2 μm/h; *n *= 9) in contrast to *Pax6*^+/+ ^(28.4 ± 3.8 μm/h; *n *= 7; Student's *t*-test: *p *= 0.0025) (Figure 2). Interestingly, however, between 2 and 6 hours post-injury, there was no significant difference in the rate of healing between *Pax6*^+/- ^(37.9 ± 4.4 μm/h) cultures compared with *Pax6*^+/+ ^(53.5 ± 12.5 μm/h; *t*-test: *p *= 0.28). The data suggest that *Pax6*^+/- ^cells experience a previously undescribed block that prevents their initial response to an injury but that they have no intrinsic cell-migration defect. We know of no other genetic defect that causes a comparable wound-healing delay.

### Calcium response to wounding

**Figure 3:**
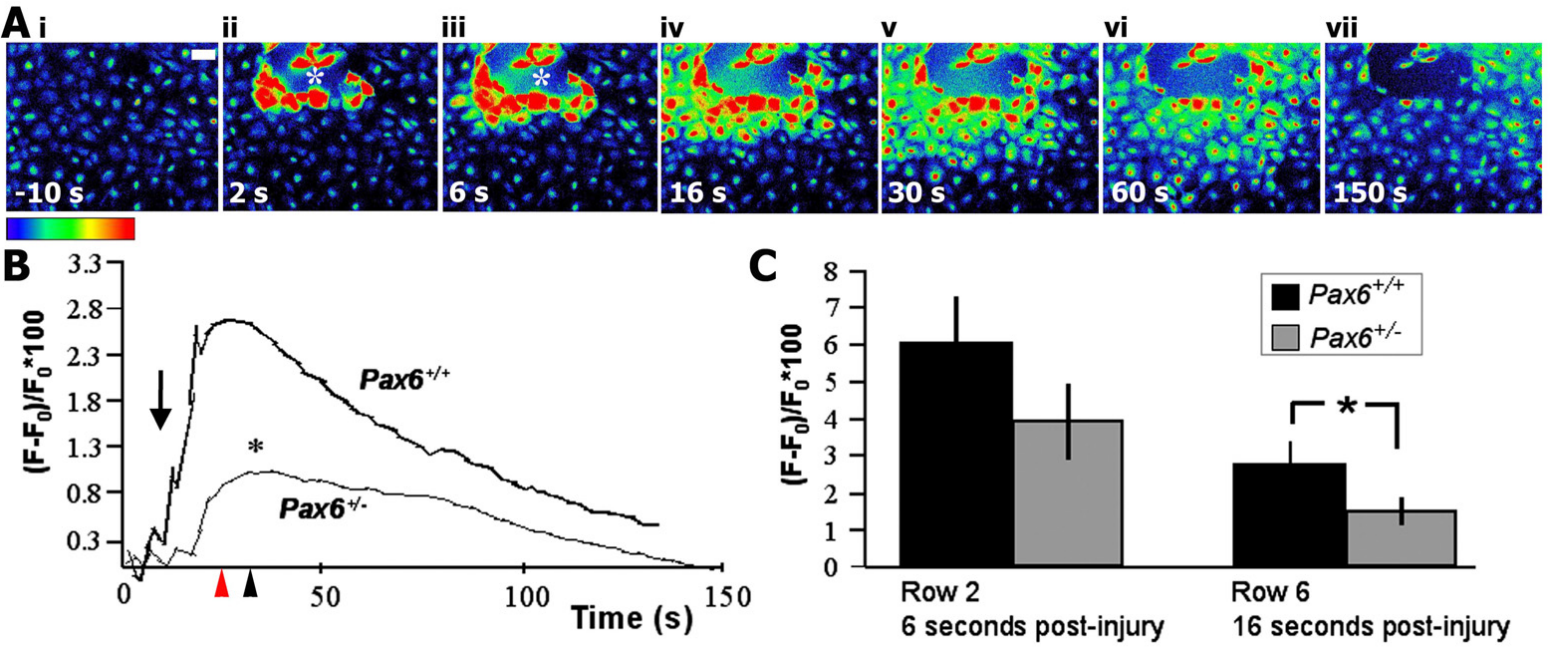
**Mechanical injury induces an intracellular Ca^2+ ^wave in corneal epithelial cultures**. **(A) **(i) Baseline image of Fluo-3 AM-loaded cells 10 s prior to injury. Scale bar = 50 μm. (ii) Initiation of a Ca^2+ ^wave 2 s after mechanical injury at point marked by *. (iii–vii) Wave propagation extended to neighboring cells outwards from the wound edge and diminished to approximate basal levels by 150 seconds. Red represents the highest and blue the lowest Ca^2+ ^concentration. **(B) **Changes in fluorescence intensity in cells six rows from the wound edge, expressed as ((F - F_0_)/F_0_) × 100 as described in Methods. The average peak fluorescence intensity was significantly reduced in *Pax6*^+/- ^cultures compared with *Pax6*^+/+ ^(*, *p *= 0.013) and occurred 12 s after injury in wild-type (red arrowhead) compared with 22 seconds in *Pax6*^+/- ^cultures (black arrowhead). Arrow, wound. **(C) **No significant difference in the intensity was observed in cell row 2 between *Pax6*^+/+ ^and *Pax6*^+/-^. Intensity in cell row 6 was significantly lower in *Pax6*^+/- ^cultures compared with *Pax6*^+/+ ^cultures (*, *p *< 0.01).

No difference was detected in the basal [Ca^2+^]_i _levels between *Pax6*^+/+ ^and *Pax6*^+/- ^cultures using Fura-2 probe (data not shown). The effects of mechanical injury on intracellular Ca^2+ ^levels were investigated using Fluo-3 fluorescent probe. Background images were acquired for each experiment (Figure 3Ai) prior to injury. Wound size was approximately 350 μm in diameter, and the wound area did not differ significantly between experiments. Immediately after injury (2 seconds), those cells directly adjacent to the wound edge exhibited a rapid rise in [Ca^2+^]_i _(Figure 3Aii). The Ca^2+ ^response propagated outwards to neighboring cells away from the wound edge (Figure 3Aiii–v). The wave began to diminish within 60 seconds, and [Ca^2+^]_i _had returned to basal levels within 3 minutes (Figure 3Avi–vii). The Ca^2+ ^response to injury was quantified in *Pax6*^+/+ ^and *Pax6*^+/- ^cultures (Figure 3B). The mean changes in fluorescence in cells in the sixth row from the wound edge were quantified over time. A significant decrease in both intensity and speed of the Ca^2+ ^wave was observed in *Pax6*^+/- ^cultures (Figure 3B,C). Interestingly, there was no significant difference in the fluorescence intensity in those cells directly adjacent to the wound between *Pax6*^+/+ ^and *Pax6*^+/- ^(row 2, 2 seconds after wounding), but the intensity of [Ca^2+^]_i _was reduced in cells in row 6, 16 seconds after injury, (Figure 3C; *p *= 0.01). The peak fluorescence intensity occurred 12 seconds after wounding in *Pax6*^+/+ ^cells but after 22 seconds in *Pax6*^+/- ^cells, and was substantially suppressed in *Pax6*^+/- ^cultures (*p *= 0.013). This is the first demonstration that changing Pax6 dosage affects calcium signaling in any system.

### Calcium wave initiation and propagation

**Figure 4:**
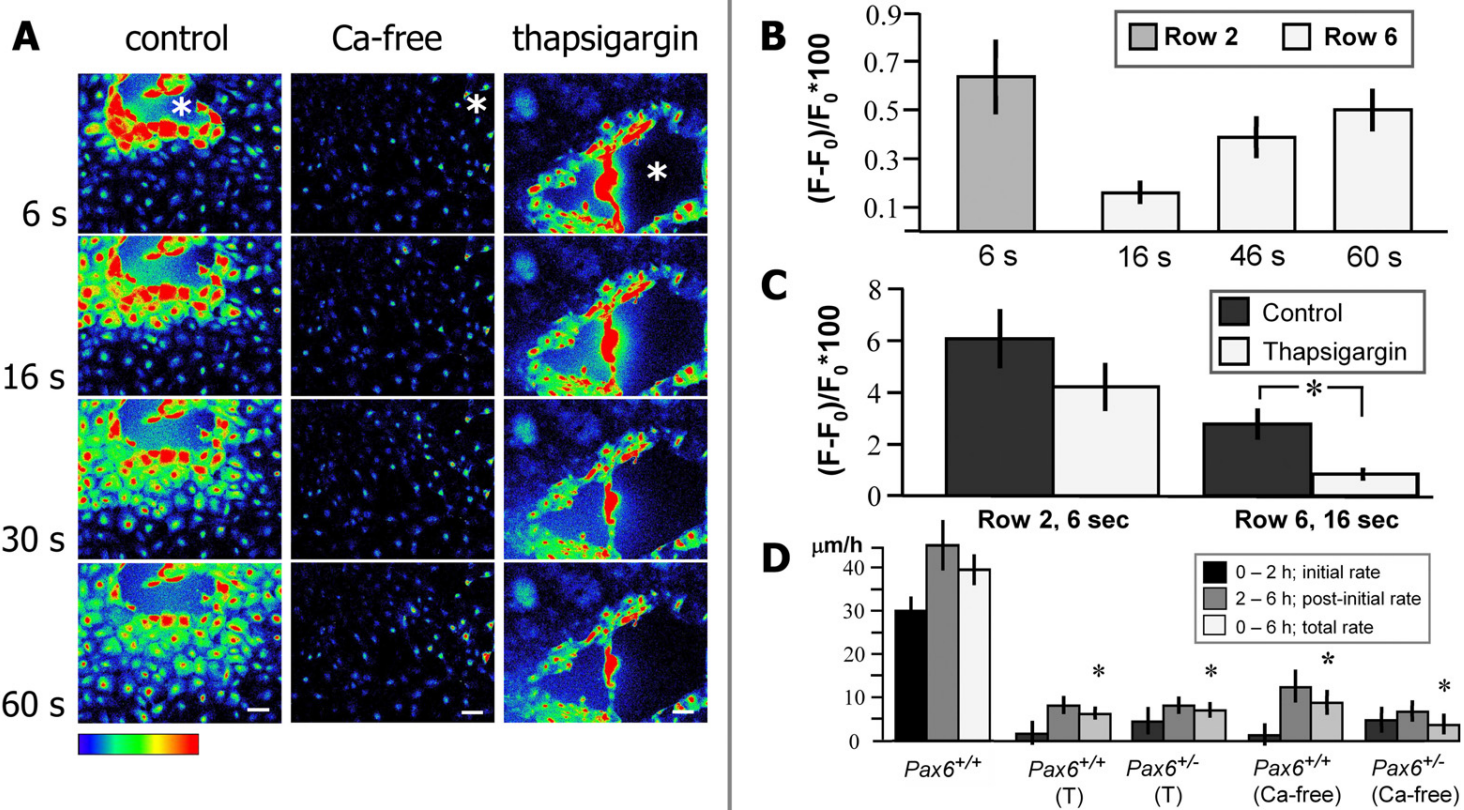
**Biphasic calcium response is required for wound healing**. **(A) **Time-lapse images show wave propagation in response to injury in control, calcium-free and 1 μM thapsigargin-treated *Pax6*^+/+ ^monolayer cultures. Removal of Ca^2+ ^from extracellular medium prevented initial rise of [Ca^2+^]_i _in cells adjacent to wound but did not prevent propagation of a significantly less intense wave. Thapsigargin did not prevent rise of [Ca^2+^]_i _in cells immediately adjacent to wound edge but inhibited wave propagation (see Additional files 1, 2, 3). **(B) **Changes in fluorescence intensity in *Pax6*^+/+ ^cultures in Ca^2+^-free HBSS in time-lapse images. **(C) **Thapsigargin-treated cultures showed no significant difference in wave initiation (row 2, 6 seconds) compared with *Pax6*^+/+^, but significantly less propagation (row 6) (*p = 0.014). **(D) **Rate of wound healing in *Pax6*^+/+ ^and *Pax6*^+/- ^cultures. Depletion of intracellular Ca^2+ ^stores with thapsigargin (T) or incubation in calcium-free medium almost completely inhibited wound healing compared with untreated *Pax6*^+/+ ^controls (*, *p *< 0.001, *n *= 6). Scale bars = 50 μm.

The initiation of the calcium wave (the immediate large calcium rise in cells surrounding the wound) was normal in *Pax6*^+/- ^cells, but the propagation was defective. We showed that the two phases can be decoupled but that both are required for healing. To investigate the source of Ca^2+ ^ions involved in the wave that spreads from the wound edge, we removed Ca^2+ ^independently from the extracellular and intracellular compartments of *Pax6*^+/+ ^epithelial cells. Cells were first injured as normal in calcium-free medium. Following injury, those cells directly adjacent to the wound edge failed to elicit a significant Ca^2+ ^response (Figure 4A). Wave propagation (at a significantly reduced intensity and slower speed) was nevertheless detectable (Figure 4B) (see Additional files 1 and 2). Peak fluorescence intensity occurred 60 seconds after injury in Ca^2+^-free media compared with 12 seconds for *Pax6*^+/+ ^cells in control media.

In separate experiments, intracellular calcium stores were depleted by 1 μM thapsigargin, which inhibits the Ca^2+^ATPase pump of the endoplasmic reticulum. Following injury, cells directly adjacent to the wound edge rapidly increased [Ca^2+^]_i_, but subsequent wave propagation was prevented (Figure 4A,C; see Additional file 3). Wave initiation and propagation in murine corneal epithelial cells can therefore be decoupled. Intracellular Ca^2+ ^is required only for wave propagation.

When either extracellular calcium or intracellular Ca^2+ ^stores were depleted, both *Pax6*^+/+ ^and *Pax6*^+/- ^cells failed to repair wounds (Figure 4D). The rate of healing in untreated *Pax6*^+/+ ^controls was 26.6 ± 1.5 μm/h compared with 6.3 ± 3.6 μm/h in thapsigargin-treated cultures (*t*-test: *p *< 0.0001) and 8.8 ± 2.9 μm/h in calcium-free media (*t*-test, *p *< 0.0001). Hence, both phases of the Ca^2+ ^response are required for coordinated wound closure.

### Propagation is via P2Y receptors, but ATP cannot rescue mutant cells

**Figure 5:**
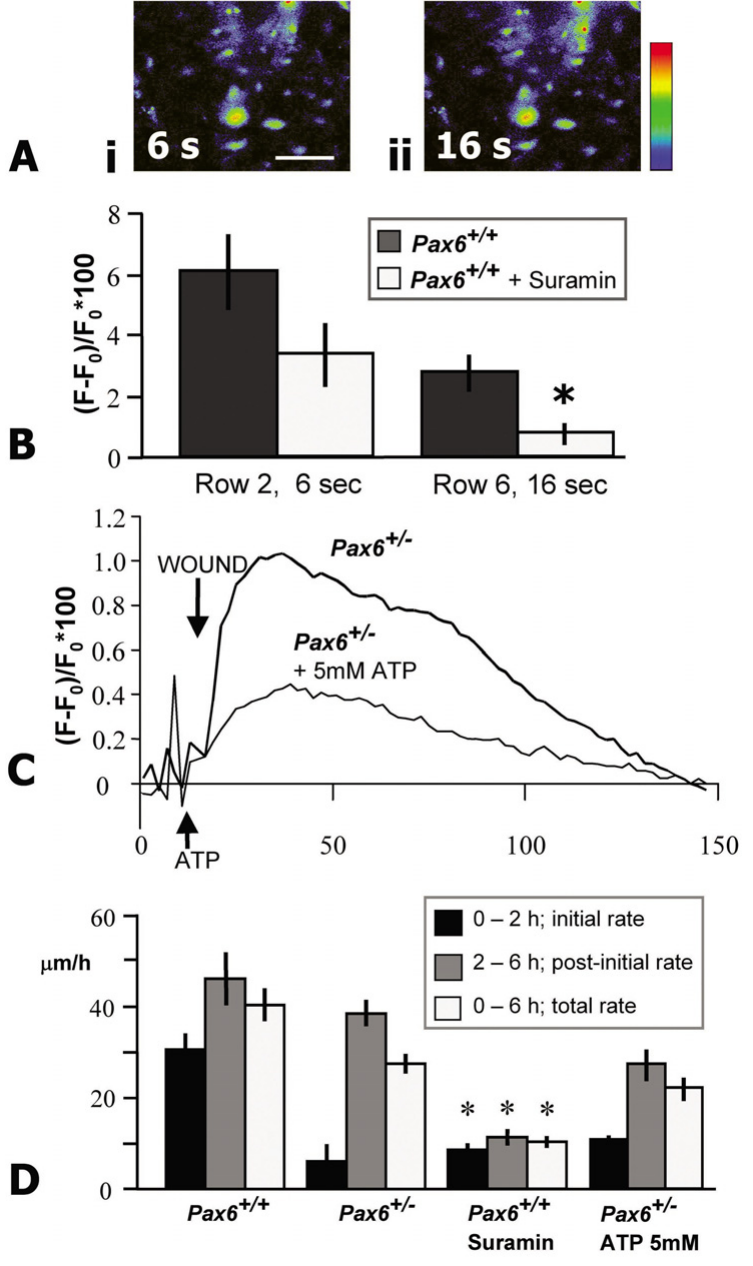
**Activation of purinergic receptors is necessary but not sufficient for wound healing of *Pax6*^+/- ^epithelia**. **(A) **Inhibition of purinergic receptors with suramin did not prevent rise of [Ca^2+^]_i _in *Pax6*^+/+ ^cells directly adjacent to the wound edge 6 seconds after injury (i), but did prevent propagation of the wave to neighboring cells after 16 seconds (ii). **(B) **No significant difference was detected in the intensities of the suramin-treated group in row 2, but intensity in row 6 was significantly reduced (*, *p *< 0.001). **(C) **ATP was added to cells 4 seconds prior to wounding (indicated by arrow). No significant difference in intensity was detected between the curves (*p *= 0.13, *n *= 12 and 9 respectively). **(D) **Suramin inhibited wound healing in *Pax6*^+/+ ^cells (*, *p *< 0.01), but ATP did not improve wound-healing rates in *Pax6*^+/- ^cells. Scale bar = 50 μm.

Adenosine triphoshate (ATP) is a potent extracellular signaling molecule involved in cell-cell communication, via purinergic (P2Y) receptors [33]. ATP can initiate a Ca^2+ ^wave, and is released in response to injury in epithelial cells [29,33]. *Pax6*^+/+ ^epithelial cells were incubated with the P2Y receptor inhibitor suramin (100 μM) prior to mechanical injury. No significant difference was detected in the Ca^2+ ^response in cells adjacent to the wound edge, but wave propagation to surrounding cells was inhibited (Figure 5A,B), suggesting a possible mechanism for wave progression in response to injury. Addition of 5 μM ATP alone could induce similar low-intensity Ca^2+ ^waves in both *Pax6*^+/+ ^and *Pax6*^+/- ^cultures, suggesting that P2Y receptors function normally in mutant cells, and that ATP release could be the missing factor in the *Pax6*^+/- ^response to mechanical injury. However, the addition of ATP 4 seconds prior to wounding in *Pax6*^+/- ^produced no improvement in the intensity (*p *= 0.13) or speed of wave progression (Figure 5C). Peak fluorescence occurred after 22 seconds in untreated *Pax6*^+/- ^cultures and after 24 seconds in those to which ATP was added. Similarly, 5 μM ATP made no impact on the rate of wound healing in *Pax6*^+/- ^cultures (Figure 5D). This suggests that ATP is not responsible for the defect in the Ca^2+ ^wave or in wound healing in *Pax6*^+/- ^cultures.

### EGF can restore the calcium wave and rescue wound-healing defects

**Figure 6:**
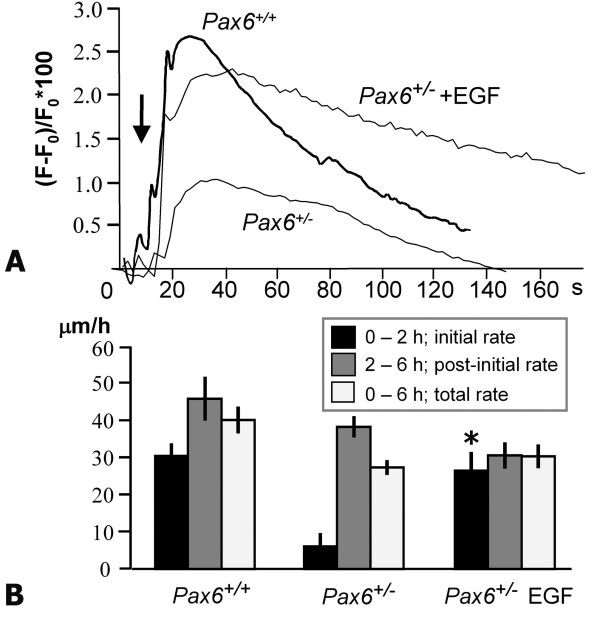
**EGF addition corrects the wound response in mutants**. **(A)**Priming *Pax6*^+/- ^cultures 90 s prior to wounding with 10 ng/ml EGF induced a significantly more intense Ca^2+ ^wave that was comparable to *Pax6*^+/+ ^cultures (*p *= 0.667). **(B) **The initial rate of healing in EGF-treated *Pax6*^+/- ^cultures was restored to wild-type levels, and significantly higher than in control *Pax6*^+/- ^cultures (*, *p *< 0.001).

EGF receptors are upregulated following corneal injury [20,44,45]. We proposed that the EGF response might mediate the rate of wound healing. We found that 10 ng/ml EGF elicited a Ca^2+ ^response in our cultures. However, the rise in *Pax6*^+/- ^cultures was delayed (mean peak 76 seconds after EGF addition versus 38 seconds in *Pax6*^+/+^) and reduced (*Pax6*^+/- ^0.087 ± 0.028% versus *Pax6*^+/+ ^0.21 ± 0.03%; *p *= 0.036, *n *= 3). *Pax6*^+/- ^cultures injured in the presence of 10 ng/ml EGF induced a wave of Ca^2+ ^of greater intensity than in control *Pax6*^+/- ^cultures, which was comparable to the control *Pax6*^+/+ ^response (Figure 6A; *p *= 0.667). Furthermore, EGF specifically improved the initial (0–2 hour) rate of wound healing in *Pax6*^+/- ^cells from 6.0 ± 3.7 μm/h to 26.5 ± 5.1 μm/h (*p *< 0.001) to a level that was comparable with *Pax6*^+/+ ^cultures (Figure 6B). Thus, the addition of EGF to *Pax6*^+/- ^cells enabled them to overcome the block that prevents them initially from responding to a wound and beginning the healing process, but did not increase the rate of cell migration in the 'post-initial' 2–6 hour phase of healing.

### ERK1/2 response to injury

**Figure 7:**
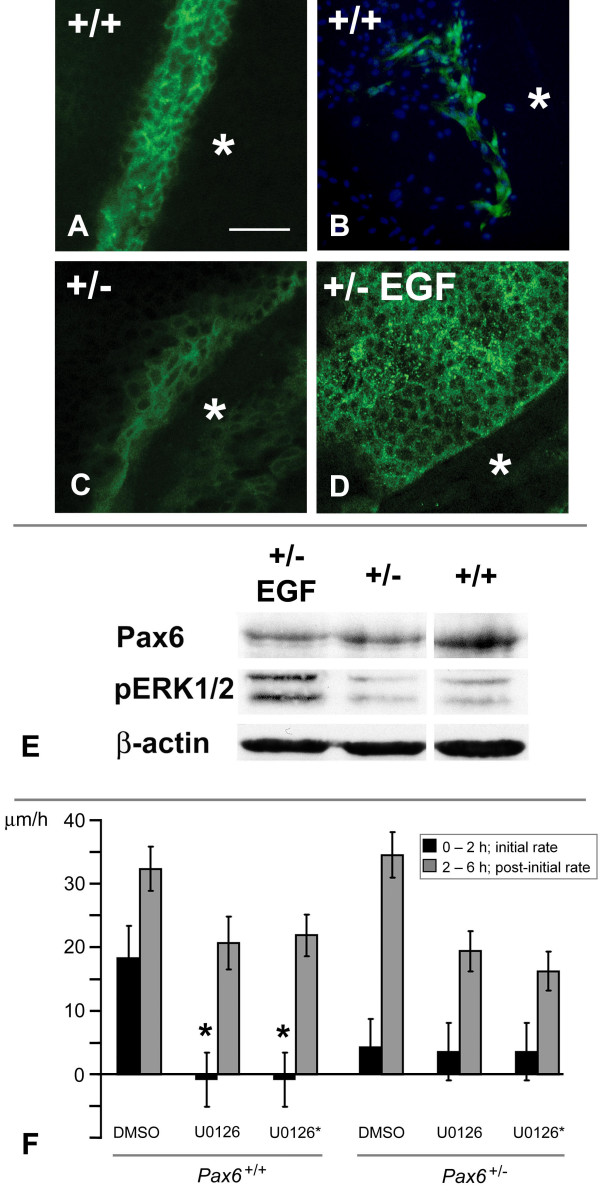
**ERK1/2 phosphorylation in wild-type and mutant cultures is required for early wound response**. **(A–D) **Immunolabeling of phospho-ERK1/2, **(A) **10 minutes after wounding in *Pax6*^+/+ ^cultures; **(B) **30 minutes after wounding in *Pax6*^+/+ ^cultures and **(C) **10 minutes after wounding in *Pax6*^+/- ^cultures. **(D) **Exposing *Pax6*^+/- ^cells to EGF 90 s before wounding elicited a large phospho-ERK1/2 response after 10 minutes. In all cases, * indicates the wounded region. Scale bars = 100 μm. **(E) **Western blots of protein lysate of *Pax6*^+/+ ^and *Pax6*^+/- ^cultures. The same blot was probed sequentially with antibodies against Pax6, phospho-ERK1/2 (P-ERK1/2), and β-actin. Left hand lane: *Pax6*^+/- ^cultures with 10 minute EGF exposure as described above. Middle lane: *Pax6*^+/- ^cultures with no EGF exposure. Right hand lane: control *Pax6*^+/+ ^cultures. Correcting for the volume of actin staining, phosphor-ERK1/2 staining in control heterozygous cultures was estimated at 80% of wild-type levels. Addition of EGF prior to wounding did not change Pax6 levels in *Pax6*^+/- ^cultures, but increased phospho-ERK1/2 staining to levels comparable with or greater than *Pax6*^+/+^. All samples were taken from pools of at least six corneal epithelial cell cultures treated identically. **(F) **Rates of wound healing of wild-type and mutant cells with addition of 10 μM U0126 (an inhibitor of phosphor-ERK1/2 signaling), 0.1% DMSO, 15 minutes prior to wounding (U), or with addition of U0126 prior to wounding and replacement of drug in fresh medium every 2 hours (U*), or *Pax6*^+/+ ^and *Pax6*^+/- ^cultures with addition of 0.1% DMSO only. (*, *p *< 0.01 compared with wild-type control).

EGF can activate the intracellular MAP kinase signaling molecules ERK1/2, which are themselves phosphorylated in response to injury [33]. Because ERK1/2 signaling has been shown to be precipitated by calcium influx in epithelial cells [46], the ERK1/2 response was investigated in our system. We found by immunocytochemistry that corneal epithelial cells showed dynamic injury-induced phosphorylation of ERK1/2 10 minutes after injury, becoming restricted primarily to the nuclei of cells at the immediate wound edge within 30 minutes (Figure 7A,B). This dynamic early wave of ERK1/2 activation, with an initially cytoplasmic localization, and variation in intensity of staining along the wound edge, is identical to that seen previously by others in the MDCK epithelial cell line [34].

*Pax6*^+/- ^cells also elicited a phospho-ERK1/2 response to wounding (Figure 7C). Using immunocytochemistry, it appeared that ERK1/2 phosphorylation in *Pax6*^+/- ^cells was stimulated by addition of EGF 90 seconds prior to wounding. This was confirmed by Western blotting (Figure 7D,E). EGF had no effect on the levels of Pax6 protein in cultures, assayed by Western blotting (Figure 7E).

We investigated the requirement for ERK1/2 phosphorylation for wound healing. Wild-type and *Pax6*^+/- ^cells were injured in the presence of 10 μM U0126 to inhibit the activated ERK1/2 pathway. It was found that addition of U0126 specifically prevented migration of *Pax6*^+/+ ^cells within the first 2 hours after wounding (*t*-test: *Pax6*^+/+ ^controls versus *Pax6*^+/+ ^U0126-treated, *p *= 0.007; *n *= 17 control, 23 drug-treated) (Figure 7C). In contrast, migration of wild-type cells 2–6 hours after wounding in the presence of U0126 was relatively normal (mean 20.7 μm/h), although still marginally significantly slower than the untreated controls (32.4 μm/h; *t*-test: *Pax6*^+/+ ^controls versus *Pax6*^+/+ ^U0126-treated, *p *= 0.039; *n *= 17 control, *n *= 15 drug-treated) (Figure 7C). To determine whether this confined effect was caused by degradation of the drug after the first 2 hours of incubation with the cells, the experiment was repeated with replacement of the culture medium with fresh medium containing 10 μM U0126 every 2 hours. This had no additional effect; the rate of *Pax6*^+/+ ^cell migration 2–6 hours after wounding with addition of fresh drug at 2 and 4 hours (21.9 μm/h, *n *= 8) was not significantly different that of experimental groups with a single addition of drug prior to wounding (20.7 μm/h, *n *= 15, as above; *t*-test, *p *= 0.81). Similarly, U0126 had no effect on wound healing by *Pax6*^+/- ^cells. In the first 2 hours after wounding, untreated cells showed negligible rates of migration, as described previously, and addition of 10 μM U0126 did not change that. During hours 2–6 after wounding, U0126-treated *Pax6*^+/- ^cells healed at 18.1 ± 3.30 μm/h, with or without addition of fresh drug – slower than untreated *Pax6*^+/- ^controls (34.5 ± 3.57 μm/h) but not significantly different from drug-treated *Pax6*^+/+ ^cells (*t*-test, *p *= 0.51) (Figure 7D).

Consistent with the transient ERK1/2 activation response to wounding, we conclude that ERK1/2 phosphorylation within the first 30 minutes is required for the initiation of cell migration. In the absence of ERK1/2 phosphorylation, *Pax6*^+/+ ^cells experience a wound-healing delay, but all cells, whatever their Pax6 genotype, can overcome this block and start migration after about 2 hours. It is clear that there are other signaling systems that can elicit a wounding response, which were not investigated here.

## Discussion

*Pax6*^+/- ^mice represent an excellent model of human *PAX6*^+/- ^aniridia-related keratopathy. Clinically, the symptoms of ARK are consistent with an altered wound-healing response [12]. Pax6 directly binds matrix metalloproteinase-9 (MMP-9, also known as *gelB*) promoter to upregulate MMP-9 in wounded corneas [47]. MMP-9 is one of a family of proteins required for tissue remodeling during wound repair. Experiments carried out previously on intact *Pax6*^+/- ^corneas suggested in a non-quantitative manner that *Pax6*^+/- ^corneas do heal [47,48], but investigated cell migration on a diseased stroma, which may modulate wound healing [49,50]. The etiology of ARK is complicated, because anterior eye development is coordinated to some extent by the lens, which is very sensitive to Pax6 dosage [51], and although the corneal epithelium expresses high levels of *Pax6 *throughout life, the stroma also expresses *Pax6 *around birth [5,52]. Corneal epithelial dysgenesis in Pax6 mutants might be the result of intrinsic defects within the epithelial cells themselves or the fault of defective tissue-tissue interactions in the eye. The extent to which tissue-tissue interactions contribute to corneal epithelial dysgenesis can be addressed by isolating the epithelial cells. If cultured mutant corneal epithelial cells were phenotypically and molecularly normal, we would infer that the corneal epithelial defects were secondary to a problem with Pax6 dosage elsewhere in the eye. To identify the primary defects that underlie the etiology of the disease, we therefore investigated wound healing in *Pax6*^+/- ^corneal epithelial primary culture. We developed and validated an *in vitro *system for wound healing in primary murine corneal epithelia. Cells formed *Pax6*-positive confluent epithelial sheets, and we demonstrated that they show physiological calcium and MAPK responses to injury. The results of the investigation were surprising, with fundamental implications for the links between transcription-factor action, cell signaling, cell behavior, and disease.

### Pax6 dosage and wound healing

*Pax6*^+/- ^corneal epithelial cultures expressed substantial quantities of Pax6, estimated to be about 60–70% that of wild-type. Pax6 autoregulates its own expression [53], and there is no direct 1:1 relationship between Pax6 gene dosage and the levels of protein present in the cells [54]. Overexpression of Pax6 in corneal epithelial cells inhibits cell-cycle progression [55], and the anterior eye is particularly sensitive to dosage of important transcription factors, with <70–80% or >~150% activity leading to dysgenesis [56]. Furthermore, corneal epithelial cells are derived from Pax6-positive stem-like cells in the corneal periphery (limbal stem cells) [57], and heterozygosity at the *PAX6 *locus compromises these cells [7] and leads to the production of corneal epithelia that may not be fully differentiated [58]. We consider, therefore, that abnormal behavior of *Pax6*^+/- ^corneal epithelial cells in culture may relate to a failure of full differentiation that occurred *in vivo *days or weeks before.

We showed that wound healing was delayed for 2 hours in mutant cells, but that once healing began, the cells moved as fast as wild-type. Rates of cell migration, at 25–50 μm/h for wild-type cells, are similar to those obtained by Song et al for wild-type rat corneas *in vivo *[43].

While this paper was under review, a new paper [59] showed that in *ex vivo *whole-eye culture, *Pax6*^+/- ^corneas healed faster than wild-type over a 24-hour period. The first 2 hours of wound healing were not studied by Ramaesh et al [59], levels of Pax6 were not assayed, and the calcium or MAPK response to wounding in the epithelium was not studied. As described above, in the whole-eye culture system, the corneal epithelial migration is modulated by the presence of an inflamed stroma, which would be expected to accelerate cell migration over extended periods of healing. Another important difference between the experiments is the size of the wounds, which were very large (1000–1200 μm) in the study by Ramaesh et al [59], encompassing up to half the cornea and encroaching on the limbal region. Larger wounds in *Pax6*^+/- ^corneal epithelia healed faster than smaller wounds (presumably because larger wounds were closer to the source of proliferating limbal stem cells), and extrapolation from figure 2 in that study [59] suggested that wounds smaller than 500 μm in diameter would heal more slowly than wild-type. Wounds in our system are smaller and more physiological, rarely larger than 200 μm, so there is therefore no immediate contradiction between our work and this new study; however, the situation appears to be more complicated than that presented by either paper, and there is clearly more work needed.

Our data show that there is no defect *per se *in the ability of mutant cells to migrate normally, providing they get the correct signals. The delay in wound healing was, however, intrinsic to the epithelia, so we investigated the immediate responses to wound healing in mutant cells.

### Calcium wave and healing rate

We are unaware of any other genetic model of epithelial wound healing where cells appear to fail to sense wounds initially, but migrate normally once this block is overcome. The mechanisms underlying this failure have wide relevance for the understanding of the molecular basis of the wound-healing response. In this study, it was found that the delay in *Pax6*^+/-^wound healing was associated with a decrease in the intensity and rate of propagation of the wound-induced calcium wave. Extracellular calcium was required for the large initial calcium rise, and intracellular calcium stores were shown to be essential for wave propagation. Hence, it is suggested that the failure in *Pax6*^+/- ^cells is due to slow or inefficient release of calcium from stores. It has been shown previously that a quantitative decrease in capacitive calcium entry to wounded gastrointestinal mucosal epithelial cells, facilitated by calcium release from the stores, leads to a quantitatively reduced healing response [31,32]. We have also found a consistent link between wound-healing rate and the intensity and speed of the calcium wave after wounding. Pharmacological disruption of any aspect of the calcium wave in *Pax6*^+/+ ^cells therefore recapitulated a wound-healing defect (Table 1). Our data strongly support a model whereby the injury-elicited calcium wave is required for wound healing [60], and indicate that the subnormal propagation of the calcium wave in *Pax6*^+/- ^corneal epithelia underlies the wound-healing delay.

**Table 1:** Calcium response, genotype and wound healing.

	High [Ca]i		Healing	
	Initiation	Propagation	0–2 h	2–6 h

***Pax6***^+/+^	**+++**	**+++**	**+++**	**+++**
No Ca^2+^_o_	-	+	-	-
No Ca^2+^_i_	+++	-	-	-
No P2Y	+++	-	-	-
+ ATP	+++	+++	+++	+++
***Pax6***^+/-^	**+++**	**+**	-	**+++**
*Pax6*^+/- ^+ ATP	+++	+	-	+++
*Pax6*^+/- ^+ EGF	+++	+++	+++	+++

+++, normal wild-type control response; +, subnormal response; -, response not detectable.

### EGF signaling and the calcium and ERK responses

In gastric mucosal epithelia, extracellular signaling (in this case, by Sonic hedgehog) elevates intracellular calcium, which in turn activates ERK, leading to cell proliferation [46]. The activation of ERK pathways by calcium signaling in response to extracellular signals has also been observed in a number of neural cell types [61-63]. Wounding of both *Pax6*^+/+ ^and *Pax6*^+/- ^corneal epithelia elicited ERK1/2 phosphorylation in a dynamic manner around the wound within 10 minutes. By 30 minutes after wounding, however, pERK1/2 immunolabeling was restricted primarily to nuclei of cells immediately adjacent to the wound edge. The pattern of immunolabeling was identical to that found previously by Matsubayashi et al [34] upon wounding of MDCK epithelial cells. They showed that inhibition of the ERK1/2 signalling pathway reduced wound healing by about 50% in the 6 hours after wounding. We also found this; over the whole 6 hours of the experiment, mean ± SEM rate of wound healing of untreated wild-type cells was 27.6 ± 2.8 μm/h, compared with 13.8 ± 2.2 μm/h for wild-type cells treated with U0126. However, we have extended the conclusions of Matsubayashi et al with a finer temporal assay of rates of wound healing, and show that although ERK1/2 inhibition does reduce the rate of cell migration during healing, ERK1/2 phosphorylation is primarily required for the rapid response to wounding within the first 2 hours.

The distribution of EGFR is abnormal in *Pax6*^+/- ^corneal epithelia [6], and our data demonstrate that the calcium reaction in response to EGF of unwounded *Pax6*^+/- ^cells is significantly lower than in *Pax6*^+/+^. Addition of exogenous EGF to wounded *Pax6*^+/- ^cells enhanced both the wound-elicited calcium wave and ERK1/2 phosphorylation, and restored the immediate migration response to wounding to wild-type levels without affecting the rate of migration. This suggests that *Pax6*^+/- ^EGF signaling is defective, perhaps due to reduced autocrine EGF production or failure of normal EGFR activity. EGF is known to enhance capacitative calcium entry (CCE) in corneal epithelial cells [39]. Because CCE is known to be quantitatively coupled with the migration speed in mucosal epithelial wound repair [31,32], this suggests a possible mechanistic explanation of positive EGF action on the wound-healing rates via calcium in our system.

EGF also acts via ERK1/2 to downregulate Pax6 and promote proliferation in rabbit corneal epithelial cells [39,64]. We detected no change in Pax6 levels following EGF addition to *Pax6*^+/- ^cells, but this is not inconsistent with the results of Li and Lu [64], because our study investigates the cellular responses within the first few minutes of wounding, whereas changes in Pax6 protein levels would be expected to take much longer than this. Furthermore, in the present study, it is unlikely that cell proliferation affects wound-healing rates, because the changes in cell proliferation take several hours (2–3 days in the study of Li and Lu [64]). We have detected no difference in 5-bromodeoxyuridine uptake between primary cultures with different Pax6 dosages, and very little cell proliferation is noted in the first 2 h after wounding (Kitazawa et al [65]; JMC, unpublished observations), whereas *Pax6*^+/+ ^cells, and *Pax6*^+/- ^cells with exogenous EGF, begin to migrate within the first few minutes. Our data suggest that EGF signaling functions via enhanced calcium and ERK1/2 signaling to initiate cell migration.

### Wound healing in *Pax6*^+/- ^corneas

Based on our data, we divide wound healing into an immediate period of 2 hours when calcium and MAPK signaling occur to initiate a rapid migratory response, and the subsequent 'post-initial' coordinated movement of epithelial sheets when cell migration is maintained. In the hours subsequent to this, cell proliferation is initiated; this last process is also modulated by Pax6 and the role of Pax6 in this regard has been studied previously [64].

We propose a model, based on our data combined with previous studies cited earlier, which postulates that *Pax6 *dosage does not affect the rate of corneal epithelial cell migration *per se *on a neutral substrate. In contrast, the primary defect of the *Pax6*^+/- ^corneal epithelium is the initial failure to respond to wounding. We have shown that upon wounding a corneal epithelium, external calcium enters cells around the wound, and this is quantitative similar in both wild-type and *Pax6*^+/- ^cells. Further propagation of calcium rise to cells away from the wound is required for the wound response, which is realized by cell-cell signaling via P2Y receptors and involves predominantly calcium from intracellular stores. We have shown that this process is inefficient in *Pax6*^+/- ^cells, and that secondary messenger ERK1/2 signaling pathways must also be activated following wounding to initiate immediate cell migration. Calcium signaling occurs in the first 2 minutes, whereas ERK1/2 signaling is expected to peak about 3–10 minutes after wounding [34]. Both calcium and ERK responses are sensitive to EGF, which is present in tears at 1–10 ng/ml [66], and we show that calcium response to EGF is diminished in heterozygotes (possibly due to mislocalisation of EGFR in the mutant epithelium [6]). Together, these cell-signaling problems decelerate the response of mutant cells to small physiological wounds. *In vivo*, the chronic delay in sealing small holes caused by minor abrasion or other cell loss may be sufficient to allow the corneal stroma to become compromised, leading to opacity. We therefore link a transcription factor mutation, via defects in calcium signaling, to a cellular abnormality that underlies human disease.

Microarray study in the lens suggested that as many as 5000 genes may be regulated by *Pax6 *[67]. It was perhaps expected therefore that the links between Pax6 and cell behavior would be pleiotropic, with a complex etiology intractable to mechanistic investigation. Our data suggest, however, that this is not the case, and that the link between genetics of disease and pharmacological correction (in this case via EGF) may not be so esoteric.

Wound healing is in part a recapitulation of important developmental pathways, [68] and it is very likely that the genetic pathways investigated here recapitulate genetic pathways that are controlled by Pax6 during development. We hypothesize that in other organ systems where Pax6 and calcium signaling are both known to be required, such as axon guidance [69,70], Pax6 dosage may directly control the kinetics of the calcium response of cells to their external environment.

## Conclusion

Propagation of wound-induced signaling via calcium release from intracellular stores is inefficient in *Pax6*^+/- ^corneal epithelial cells, and this underlies an intrinsic wound-healing delay. Pax6 dosage does not, however, affect the rate of cell migration in healing epithelia, 2–6 hours after wounding. MAP kinase signaling via phosphorylation of ERK1/2 is required for the immediate wound-healing response, but has only a marginal effect on the rate of cell migration in a healing epithelium. Addition of EGF prior to wounding rescues the calcium response in mutant cells and exaggerates the levels of ERK1/2 phosphorylation, restoring rapid wound-induced cell migration. Failure to respond quickly to minor wounding in fragile *Pax6*^+/- ^corneal epithelia could lead to the observed chronic corneal degeneration in patients, but our data suggest that pharmacological manipulation of the EGF pathway is a potential therapeutic strategy.

## Methods

### Mice and cell culture

*Pax6*^+/*Sey*-*Neu *^(*Pax6*^+/-^) mice [71] were maintained on the CBA/Ca genetic background by heterozygous mating. Corneas were dissected from *Pax6*^+/+ ^and *Pax6*^+/- ^littermates 8–10 weeks old. A small proportion of heterozygous corneas were rejected for use because they exhibited severe ocular surface damage. Previous work had demonstrated that adult murine *Pax6*^+/- ^corneal epithelia are cytokeratin-12-positive with only minor incursion of goblet cells, and no other conjunctival contamination was detected [58].

Studies were performed using primary monolayer corneal epithelial cells. Corneas were isolated and cultured according to the method of Hazlett et al [40]. Central corneal buttons, free of conjunctival cells, were washed in phosphate-buffered saline (PBS) containing 500 U/ml penicillin G and 500 μg/ml streptomycin for 10 minutes and transferred to 35-mm plastic dishes or glass coverslips as appropriate, epithelium-side up, for 5 minutes before addition of complete culture medium (15 ml keratinocyte basal medium (Cambrex, UK), 19 ml DMEM:F12, 5 ml fetal bovine serum (12.5% v/v), 125 μg/ml gentamycin, 125 μM 2-β-mercaptoethanol and 25 mM HEPES). The corneal explant was removed after 14 days and cultures were used 2–3 days later. Corneal epithelial cells cultured in this way have previously been shown proteomically to be very similar to fresh corneal epithelia, and express major corneal epithelial markers [40].

### Chemicals and reagents

Thapsigargin and suramin were from Calbiochem (Nottingham, Nottinghamshire, UK); MEK inhibitor U0126 from Promega (Southampton, Hampshire, UK); EGF and ATP from Sigma-Aldrich (Poole, Dorset, UK); anti-phospho-ERK1/2 from NE Biolabs (Hitchin, Hertfordshire, UK). Anti-Pax6 monoclonal antibody developed by A. Kawakami was obtained from the Developmental Studies Hybridoma Bank developed under the auspices of the NICHD (University of Iowa, IA, USA). Fluorescent probes were from Invitrogen (Paisley, UK).

### Ca^2+ ^imaging

Calcium imaging was performed in modified Hanks' balanced salt solution (mHBSS; 2.5 mM CaCl_2_, 150 mM NaCl, 4 mM KCl, 1 mM MgCl_2_, 10 mg/ml D-glucose, 20 mM HEPES). For experiments in Ca^2+^-free medium, the CaCl_2 _was replaced with 1 mM EGTA.

Murine corneal epithelial cells grown on glass coverslips were incubated for 45 minutes in mHBSS with 8.8 μM Fluo-3 AM in dimethyl sulfoxide (final concentration 0.2%) and Pluronic F-127 (final concentration 0.02%). Drugs were preincubated with the cells in mHBSS during the Fluo-3 incubation. Cells were washed twice with mHBSS (Ca-free or with drugs when appropriate) and positioned on a Zeiss Axiovert S100T microscope with a 100-W mercury arc lamp, a cooled CCD camera (Quantix, Photometrics) and 10 × objective. For excitation, an FITC filter (480/30 nm) was used. No emission filter was used. Images were captured at least 15 seconds before injury (an approximately circular wound using a 3G needle) to establish the baseline of fluorescence reading at 2-second intervals for 180 seconds. For experiments in which EGF was added prior to wounding, imaging started 70 seconds after EGF addition, and the cells were injured after further 20 seconds.

### Image analysis

Ca^2+ ^dynamics were evaluated using the changes in fluorescence intensity of calcium probe Fluo-3. MetaMorph (Universal Imaging Corp.) and Image J  software were used. The data are presented as percentage change in the fluorescence intensity at each time point (F) to the first time point (F_0_) reading; the term ((F - F_0_)/F_0_) × 100 was used to represent this percentage change. In captured images, the second and sixth cell rows from the wound edge were drawn. The second-row intensity was assessed in order to study the calcium dynamics in cells adjacent to the wound. The first cell row on the wound edge was not evaluated because it included damaged cells. The fluorescence intensity of the sixth cell row was used to investigate the spread of calcium waves from the wound edge into the neighboring tissue.

### Rate of wound healing in epithelial cultures

Small wounds were introduced into confluent *Pax6*^+/+ ^and *Pax6*^+/- ^monolayers in 35-mm dishes using a fire-polished glass pipette. The initial wound size and shape were similar and therefore shared the same mechanistic features in healing. The distance across each wound in four places was measured using calibrated eyepiece graticules every hour for 6 hours or until wounds were completely closed. Cells were incubated at 37°C, 5% CO_2 _at all times. Rate of wound healing was expressed in μm/h.

For inhibition of phospho-ERK1/2 signaling, 10 μM U0126 (made up as a 10 mM solution in DMSO) was added to the cells in culture 15 minutes before wounding unless stated otherwise.

### Immunocytochemistry

Cultures were fixed in PBS containing 4% w/v paraformaldehyde, permeabilised in methanol for 10 minutes at -20°C, and rinsed three times with PBS before blocking for 30 minutes (blocking buffer: 0.3% BSA, 4% normal goat serum, PBS). Cells were incubated in primary antibody (Pax6 diluted 1:50, Phospho-ERK1/2 diluted 1:300, both in blocking buffer) overnight at 4°C. After three 5-minute washes in PBS, secondary antibody (Alexa fluor 488 goat anti-mouse, 1:200 in blocking buffer) was added for 1 hour. Following three 5-minute washes in PBS, cells were mounted in Vectashield (Vector Laboratories).

### Statistical analysis

Unpaired Student's *t*-test was used for assessment of significance of differences between groups. The non-parametric Mann-Whitney test was used when the standard deviations of compared groups were significantly different.

## Authors' contributions

LJL and PW carried out the cell cultures, wounding assays, and all measurements of calcium and ERK responses, and interpreted the data. LJS tested the wound-healing response in presence of ATP and suranim. JO, RK and DL developed the primary cell culture techniques and the wounding assays and performed Western blots. JVF, CDM and MZ helped draft the manuscript and participated in design of the study. JMC conceived the study, participated in its design and coordination, performed experiments and helped to draft the manuscript. All authors read and approved the final manuscript.

## Supplementary Material

Additional file 1 **Calcium signalling in control wounded corneal epithelium **Fluo-3 imaging of intracellular calcium after wounding. Total length of video represents 2 minutes, red represents the highest calcium concentration, blue/violet the lowest. The wave initiates strongly at the wound edge and travels into the epithelial sheet. Click here for file

Additional file 2 **Calcium signalling in Pax6^+/+ ^corneal epithelium wounded in calcium-free medium **Fluo-3 imaging of intracellular calcium after wounding in calcium-free HBSS. Total length of video represents 2 minutes, red represents the highest calcium concentration, blue/violet the lowest. There is no strong calcium response immediately adjacent to the wound edge, but a weak calcium wave is visible that propagates from the wound edge into the tissue. Click here for file

Additional file 3 **Calcium signalling in Pax6^+/+ ^corneal epithelium wounded in the presence of thapsigargin. **Fluo-3 imaging of intracellular calcium after wounding. Total length of video represents 2 minutes, red represents the highest calcium concentration, blue/violet the lowest. The wave initiates strongly at the wound edge but fails to propagate into the epithelial sheet. Click here for file
